# Effectiveness of integrated nurse-physician management in post-PCI coronary heart disease patients: an analysis based on follow-up data

**DOI:** 10.3389/fcvm.2025.1571953

**Published:** 2025-10-14

**Authors:** Xiaojing Qiu, Songzeng Ren

**Affiliations:** ^1^ School of Nursing, Henan Technical Institute, Zhengzhou, Henan, China; ^2^ Interventional Operating Room, The Third People’s Hospital of Henan Province, Zhengzhou, Henan, China

**Keywords:** coronary heart disease, PCI, integrated care, secondary prevention, quality of life, patient education

## Abstract

**Background:**

Post-PCI patients often require coordinated secondary prevention and education. We evaluated whether an integrated nurse–physician management program improves health behaviors, disease knowledge, angina-related health status, and cardiac function vs. routine care.

**Methods:**

In a single-center, retrospective, non-randomized cohort, adults after PCI received either an integrated program or routine care. Outcomes were assessed at baseline, 1 month, and 3 months and included: Health-Promoting Lifestyle Profile II (HPLP-II), Coronary Artery Disease Education Questionnaire—Short Version (CADE-Q SV), Seattle Angina Questionnaire (SAQ-overall), echocardiographic indices (e.g., LVEF, LVFS), and physiological/biochemical markers (SBP, DBP, BMI, triglycerides, LDL). The primary endpoint was change in SAQ-overall from baseline to 3 months. Linear mixed-effects models with fixed effects for group, time, and group × time and a subject-level random intercept estimated estimated marginal means (EMMs) and the between-group difference-in-change (*ΔΔ*) with 95% CIs. Multiplicity was controlled using Holm adjustment within prespecified outcome families. Per-timepoint Welch's *t*-tests and within-group paired t-tests served as sensitivity analyses.

**Results:**

128 patients were analyzed (64 integrated; 64 routine). At 3 months, the integrated program produced a significantly greater improvement in SAQ-overall vs. routine care (*ΔΔ* favoring integrated). HPLP-II and CADE-Q SV also improved more with the integrated program, and echocardiographic indices showed a significant group × time effect consistent with better recovery in the integrated group. Physiological/biochemical markers exhibited mixed patterns: both groups showed within-group reductions in SBP/DBP, triglycerides, and LDL, while BMI decreased modestly and non-significantly in the observation group but reached significance in the control group. Findings were directionally robust in sensitivity analyses.

**Conclusions:**

Over 3 months post-PCI, integrated nurse–physician management improved patient-reported outcomes and cardiac function beyond routine care, while changes in physiological/biochemical markers were variable. These results support integrating structured nursing-led secondary prevention and education into routine post-PCI management and warrant confirmation in prospective randomized studies.

## 1 Introduction

Cardiovascular disease (CVD) remains the leading cause of death worldwide, accounting for approximately 32% of all deaths in 2022 (1). The latest Global Burden of Disease (GBD) analyses likewise show that ischaemic heart disease has long ranked as the single leading cause of death globally (2). Percutaneous coronary intervention (PCI) is a principal therapy to relieve vascular stenosis and improve clinical symptoms; however, PCI provides only symptomatic relief and does not eliminate the underlying atherosclerotic pathology. Long-term antithrombotic therapy is therefore required after the procedure, which poses substantial demands on patients’ self-management (3). Meanwhile, patients with coronary heart disease often maintain unhealthy lifestyle habits even after surgery; disruption of the physiological–pathological trajectory thereby increases the risk of in-stent restenosis (4–6). Evidence indicates that the 3–6 months after the procedure constitute a critical period of ongoing disease progression (7). Accordingly, it is essential to modify unhealthy behaviors and strengthen patients’ self-management. Prior research suggests that integrated nurse–physician management models can help slow disease progression and promote recovery (8, 9). On this basis, drawing on the literature, clinical experience, and patient preferences, we established a specialized nursing team to enhance interprofessional collaboration, aiming to improve self-management after PCI, facilitate the abandonment of detrimental habits, enhance cardiac function, and improve quality of life.Although previous reports commonly define the first 3–6 months after PCI as a vulnerable window for adverse events and symptom fluctuation (7), we deliberately assessed outcomes at 1 month and 3 months for three reasons. First, the initial 30 days and the subsequent 12 weeks correspond to routine post-PCI medication titration and Phase II (outpatient) cardiac rehabilitation, during which health behaviors, disease knowledge, and angina-related quality of life are most responsive to integrated, multidisciplinary management. Second, the 3-month visit lies at the lower bound of the 3–6-month window, allowing early trajectory detection while reducing attrition and contamination that accumulate by 6 months in real-world practice. Third, because this analysis used retrospectively assembled service data during program roll-out, complete and balanced 6-month capture was not available for all patients; restricting analyses to 1 and 3 months minimized informative censoring and preserved internal validity. Accordingly, we interpret 1- and 3-month changes as early surrogates within this critical period and plan prospective extensions to 6 and 12 months to confirm durability of effects.

## 2 Materials and methods

### 2.1 General information

This was a single-center, retrospective, non-randomized cohort conducted at a tertiary cardiovascular hospital in Henan, China. During routine service rollout, an integrated nurse–physician management program for post-PCI care was introduced as a clinical service (not a trial). Patients who received the integrated program constituted the observation group, whereas contemporaneous patients managed under routine care formed the comparison group. Exposure (integrated vs. routine) was identified retrospectively from service logs and electronic health records (EHRs); no protocolized randomization occurred. Participants and allocation: Adults (≥18 years) with coronary heart disease treated with PCI were eligible if they were clinically stable at discharge, could complete questionnaires, and had baseline plus ≥1 follow-up assessment (1 or 3 months). We excluded patients with hemodynamic instability; severe cognitive/psychiatric disorders precluding valid self-report; end-stage renal/hepatic failure; or a scheduled staged PCI within 3 months. Diagnosis and eligibility were determined according to the most recent international standards, specifically the 2025 ACC/AHA/ACEP/NAEMSP/SCAI Guideline for Acute Coronary Syndromes (10).Allocation reflected care-as-received (non-random). To address selection bias, we provide a comprehensive baseline characteristics table reporting standardized mean differences (SMDs) alongside *p*-values, and we prespecified length of hospital stay (LOS) as a contextual covariate given its potential association with recovery and service uptake.Usual care vs. integrated program (definitions):Routine care comprised ward-based discharge education, medication reconciliation with written instructions, and cardiology visits at approximately 1 month and 3 months, with *ad hoc* telephone checks per unit policy. The integrated program added structured risk-factor counseling, symptom monitoring and medication-titration support, and team-based education via clinic contacts and WeChat messaging on a predefined schedule. Full TIDieR details (who/what/where/when/how much/tailoring/fidelity) are provided in the Intervention subsection. Sampling frame and timeframe. We retrospectively identified 128 post-PCI patients admitted December 2023–January 2024: integrated/observation *n* = 64 and routine/control *n* = 64. All records were reviewed using standardized retrospective procedures. Follow-up timing (for consistency with reviewers):Follow-up windows were predefined as 28–35 days (1 month) and 84–98 days (3 months) to align with post-PCI clinic schedules and early rehabilitation milestones.

### 2.2 Care methods

Care was delivered at a tertiary cardiovascular specialty hospital in Henan, China. Post-discharge follow-up was primarily hospital-based and coordinated by the cardiology service, with scheduled clinic visits at approximately 1 month and 3 months, supplemented by standardized telephone/WeChat check-ins. Formal Phase II cardiac rehabilitation was not routinely available during the study period; patients received exercise and lifestyle counseling within either routine care or the integrated program. Community/primary-care follow-up was not protocol-mandated and occurred at the discretion of patients and local providers.

Routine management consisted of standard care delivered within the hospital pathway and usual outpatient clinics. Who provided: bedside ward nurses and attending/fellow cardiologists during hospitalization; outpatient cardiologists and clinic nurses after discharge. What it included: (i) ward-based discharge education covering dual antiplatelet/statin/β-blocker/ACEI/ARB adherence, risk-factor targets (blood pressure, LDL-C, glucose), smoking cessation, diet, weight control, graded physical activity and return-to-work advice, and recognition of warning signs with instructions on when to seek care; (ii) medication reconciliation with written instructions and a printed follow-up plan; (iii) scheduled cardiology visits at approximately 1 month and 3 months; and (iv) *ad hoc* telephone/WeChat contacts triggered by patient request, abnormal results, or clinician discretion. Context/setting: initiation on the inpatient ward prior to discharge, followed by usual care in the hospital's cardiology clinics. What it did not include: no protocolized Phase II cardiac rehabilitation, no structured remote coaching schedule, no proactive team-based WeChat education, and no protocolized home visits. Community/primary-care follow-up was not mandated and occurred at patient/local-provider discretion.

Brief name&rationale: Integrated nurse–physician management for post-PCI secondary prevention to enhance self-management, symptom control, and timely medication titration.Who provided: A multidisciplinary team comprising a chief cardiologist, attending cardiologists, cardiovascular nurse specialists and dedicated ward/clinic nurses, a rehabilitation physician, and a psychological counselor; all members had ≥5 years of clinical experience. All team members completed standardized training and passed competency assessments based on the Self-management Education Manual for Postoperative Patients with Coronary PCI, and were trained in patient education and secondary-prevention management. The same team was responsible for initiating the intervention during hospitalization and for post-discharge follow-up, ensuring continuity and consistency of care. What (materials & procedures).The intervention comprised individualized counseling on adherence to antiplatelet/β-blocker/statin therapy, control of BP/glucose/lipids, diet, smoking cessation, and graded physical activity; proactive symptom monitoring (angina frequency, dyspnea, BP/HR logs) with predefined escalation thresholds (e.g., rest angina; SBP <90 or >180 mmHg; HR <50 or >110 bpm); physician-led medication titration with nurse follow-up; educational micro-modules and reminders delivered via WeChat (texts/short videos/posters); and a printed take-home plan (warning signs, contacts, follow-up dates). The psychological counselor explained how negative emotions impede recovery and provided brief coping guidance, escalating when red-flags emerged; the nurse-in-charge reinforced evidence-based diet and smoking cessation with practical adherence steps; the rehabilitation physician distinguished “rational” from “irrational” exercise, prescribed progressive aerobic training, and reiterated safety thresholds; and the attending cardiologist addressed patient questions on the expected recovery trajectory and prevention of post-PCI complications. Intervention fidelity was ensured through adherence to departmental SOPs, standardized scripts, and checklists across all team members. Where: Initiated on the inpatient ward before discharge; continued via outpatient clinics and remote (telephone/WeChat) contacts. When & how much (dose): One 30–40 min pre-discharge session; after discharge, weekly contacts during weeks 1–4, then biweekly during weeks 5–12; clinic visits at ∼1 month and ∼3 months. Each remote contact typically 10–15 min; messages acknowledged within 24 h on working days. (Adjust to your actual cadence if needed.)Tailoring: Content adapted to baseline knowledge, literacy, comorbidities (e.g., diabetes/hypertension), functional status, caregiver availability, and patient preferences; exercise plans risk-stratified and progressed accordingly. Modifications: No protocol changes during the study period. Fidelity (provider adherence): Use of standardized scripts/checklists; all planned contacts recorded in a log; monthly audit by the lead nurse (10% random sample) verifying timing/content against the protocol and EHR notes. Adherence (patient engagement) & feasibility metrics (through 3 months). Completed contacts/planned contacts, %: Integrated (observation): 456/512 (89.1%). Routine (control):(no protocolized remote schedule).Clinic attendance:1-month visit: Integrated 61/64 (95.3%); Routine 57/64 (89.1%). 3-month visit: Integrated 59/64 (92.2%); Routine 55/64 (85.9%). WeChat engagement (integrated program only).Read rate: 92%. Response rate to prompts: 85%.Attrition (loss to follow-up by 3 months). Integrated: 4/64 (6.3%).Routine: 7/64 (10.9%). Escalations triggered (alerts leading to clinician review), n Integrated: 9.Routine: 6.

Questionnaires were administered by trained nurses who were not blinded to group assignment; however, data abstractors and analysts were blinded to the study hypotheses.

### 2.3 Observation indexes

#### 2.3.1 Health promotion lifestyle profile-II (HPLP-II)

Used to assess participants’ health promotion lifestyles. Originally developed by Walker in 1987 with a Cronbach alpha coefficient of 0.922, indicating high reliability. Revised by Wenjun Cao et al. in 2016 for community populations, with coefficients ranging from 0.630 to 0.810.Comprises 40 items across six dimensions, scored on a 4-point Likert scale, with higher scores indicating better health-promoting behaviors. In this study, the Cronbach alpha was 0.933, confirming reliability (11).

#### 2.3.2 Coronary artery disease education questionnaire short version (CADE-QSV)

Developed by GHISI et al. in 2016 and revised by Li Jiajia in 2020 to assess knowledge in coronary artery disease patients. Includes 20 items, with higher scores indicating better knowledge.In this study, the Cronbach alpha coefficient was 0.77 (12).

#### 2.3.3 Seattle angina questionnaire (SAQ)

Revised by Spert et al. in 1994 to evaluate angina-related quality of life. Comprises 27 items, with higher scores indicating better quality of life. In this study, the Cronbach alpha was 0.89, indicating strong reliability (13).

#### 2.3.4 Biochemical indicators

Recorded before and after the intervention, including systolic blood pressure (SBP), diastolic blood pressure (DBP), fasting blood sugar (BG), body mass index (BMI), total cholesterol (TC), triglycerides (TG), low-density lipoprotein (LDL), and high-density lipoprotein (HDL).

### 2.4 Data collection

Baseline questionnaires were self-administered under nurse supervision pre-discharge. Follow-ups at 1 month (28–35 days) and 3 months (84–98 days) were conducted via in-person clinic visits or standardized telephone/WeChat contacts using uniform forms. Clinical and laboratory data were abstracted from the EHR with a predefined dictionary. Data abstractors were blinded to study hypotheses and trained on the protocol; 10% of records underwent double entry for quality control.

### 2.5 Statistical analysis

For each continuous outcome, we fit a linear mixed-effects model (LMM) with group (observation vs. control), time (baseline, 1-month, 3-month), and the group × time interaction as fixed effects, and a subject-level random intercept to account for within-patient correlation. We report estimated marginal means (EMMs) at each time point, the between-group difference-in-change (*ΔΔ* = *Δ*_observation−*Δ*_control) with 95% confidence intervals (CIs), and standardized effect sizes (Hedges’ g). Model assumptions (residual normality and homoscedasticity) were checked; two-sided *P* values are presented and Holm adjustment was applied within prespecified outcome families.When baseline imbalance was present (SMD > 0.10 or *P* < 0.20), we prespecified inclusion of age, sex, LVEF, and NYHA class as covariates. Parameters were estimated by REML, and degrees of freedom were computed using the Satterthwaite approximation. Skewed biochemical variables (e.g., triglycerides)were log-transformed for modeling and back-transformed for presentation when appropriate. The primary endpoint was the change in SAQ-overall from baseline to 3 months. Secondary endpoints included HPLP-II, CADE-Q SV, echocardiographic measures (e.g., LVEF, LVFS), and physiological/biochemical markers (SBP, DBP, BMI, triglycerides, LDL). For each endpoint we present EMMs, within-group change (*Δ*), and *ΔΔ* with 95% CIs; multiplicity was controlled by Holm correction within families (patient-reported outcomes; echocardiography; physiological/biochemical markers). The single primary endpoint was not additionally adjusted for multiplicity. The significance level was *α* = 0.05 (two-sided).Sensitivity analyses comprised: (1) per-timepoint between-group contrasts using Welch's *t*-tests with Hedges’ g; (2) within-group paired t-tests for pre-to-post changes; and (3) a complete-case analysis alongside the primary LMM. Missing data were assumed missing at random (MAR), under which LMMs provide valid inference; for endpoints with >10% missingness, we additionally performed multiple imputation by chained equations (m = 20) and re-fit the primary models. We also report the minimal detectable effect (MDE) for the primary endpoint at *α* = 0.05% and 80% power, based on the observed variance–covariance structure. All analyses were performed in IBM SPSS Statistics 27.0 and R 4.3.1 (packages: lme4, lmerTest, emmeans, multcomp), adhering to STROBE and TIDieR reporting guidance.

## 3 Results

### 3.1 Comparison of HPLP-II scores at different time points between the two groups

**Figure 1:**
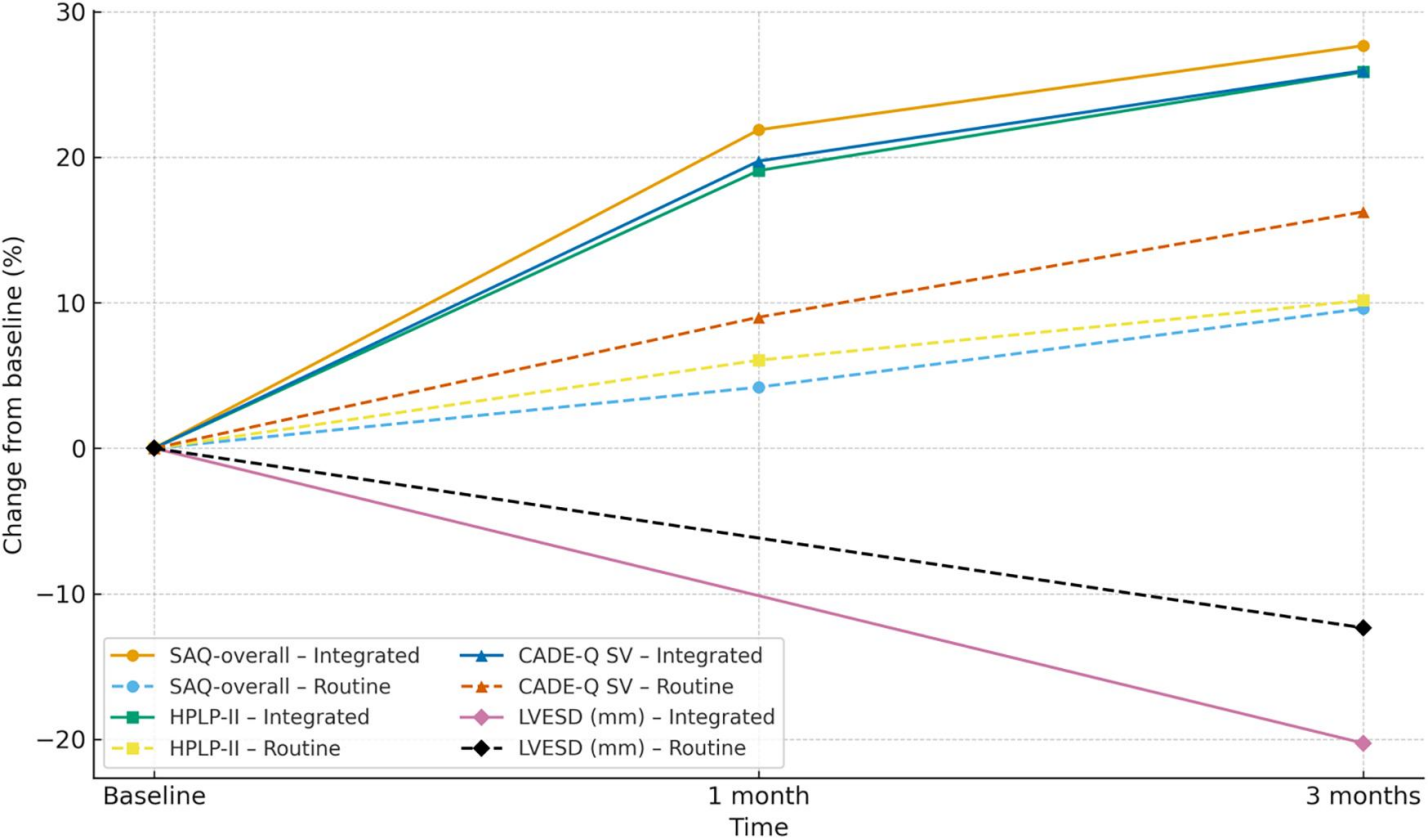
Spaghetti plot of HPLP-II scores over time in the two groups. Each thin line represents an individual patient's score across baseline, 1 month, and 3 months. Blue lines represent the observation group; orange dashed lines represent the control group. Bold lines indicate group means.

The HPLP-II scores in both the observation and control groups demonstrated an upward trend after 1 and 3 months of intervention. Notably, the increase in HPLP-II scores within the observation group was significantly higher than that observed in the control group (*P* < 0.001), as illustrated in Table 1; Figure 1.

**Table 1:** Comparison of HPLP-II scores between the two groups of patients (scores. $\bar{x}\pm s$).

Outcome	Integrated *n* = 64	Routine *n* = 64	Mean difference(integrated -routine)	95% CI of difference	Welch t	*P*	Hedges’ g	95%CI to g
Baseline	107.65 ± 11.52	108.23 ± 11.12	−0.58	−4.15 to 2.99	0.32 (125.0)	0.752	−0.05	−0.40 to 0.29
1 month	128.19 ± 9.35	114.78 ± 9.66	+13.41	10.53 to 16.28	8.55 (125.7)	<0.001	1.40	1.02 to 1.79
3 months	135.49 ± 9.66	119.23 ± 10.46	+16.26	13.09 to 19.42	9.93 (124.7)	<0.001	1.61	1.21 to 2.00
F	Fgroup = 170.601	Ftime = 317.500	Fgroup × time = 17.570					
*P*	<0.001	<0.001	<0.001					

Values are mean ± SD unless stated. Primary analysis used linear mixed-effects models (group, time, group × time) with Holm adjustment for multiple comparisons; per-timepoint Welch *t*-tests are shown as sensitivity analyses. Mean difference is Integrated -Routine. Hedges’ g is reported with 95% CI. Two-sided *α* = 0.05.

### 3.2 Comparison of CADE-QSV scores at different time points between the Two groups

**Figure 2:**
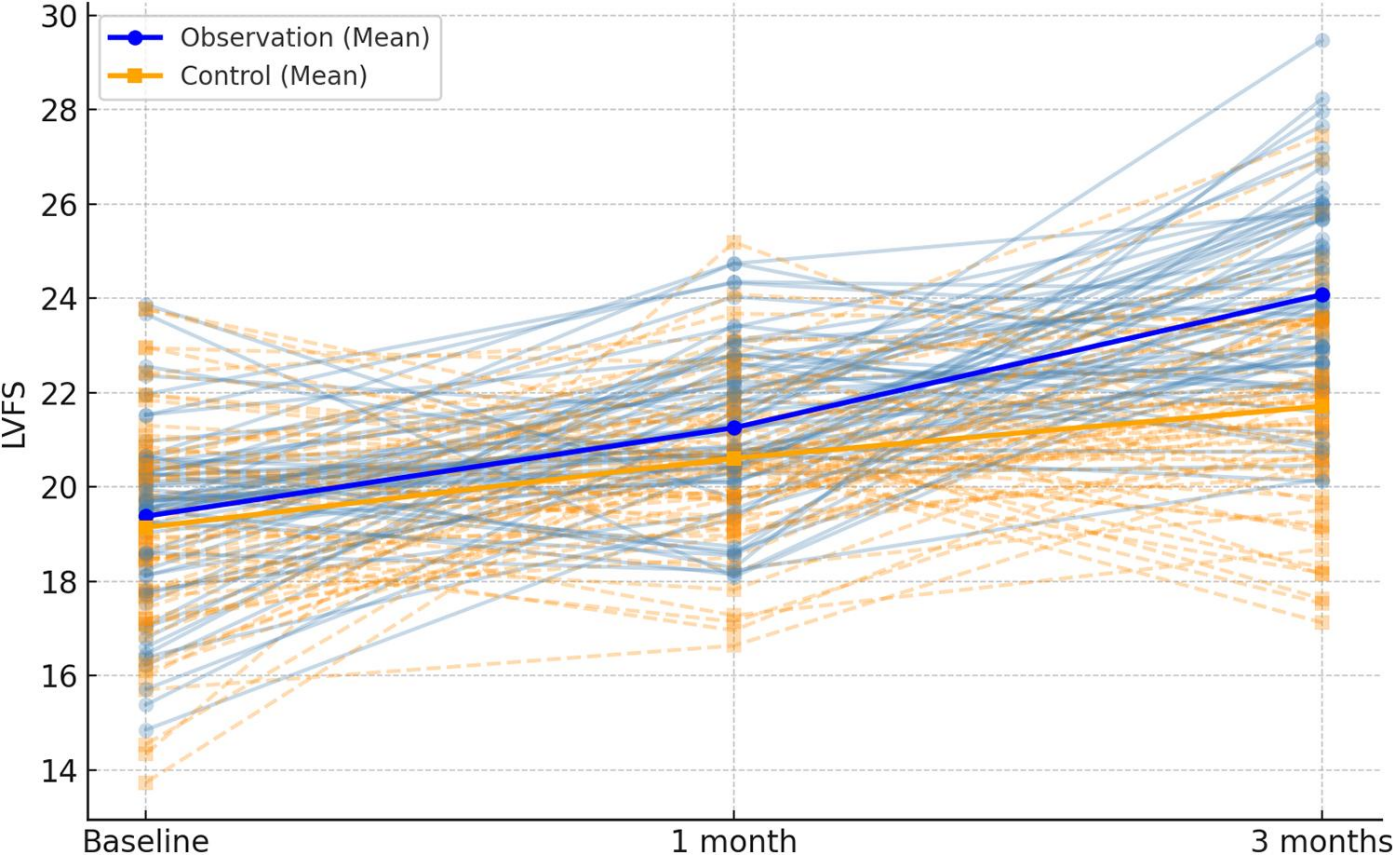
Spaghetti plot of CADE-QSV scores across the follow-up period. Individual patient trajectories are shown as thin lines, with observation group in blue and control group in orange. Mean values are overlaid as bold lines.

In this study, baseline characteristics were assessed for the two groups (routine care and integrated program), and as shown in Table 2, there were no statistically significant differences between the groups (*P* > 0.05). The CADE-QSV scores for both the observation and control groups exhibited a notable increase after 1 month and 3 months of intervention. Significantly, the increase in CADE-QSV scores within the observation group was substantially higher than that observed in the control group (*P* < 0.001), as detailed in Table 3; Figure 2.

**Table 2:** Baseline characteristics of the cohort.

Characteristic	Routine care (*n* = 64)	Integrated program (*n* = 64)	SMD	*P*
Age, years	62.40 ± 8.10	61.80 ± 8.30	0.07	0.68
Female, *n* (%)	21 (33.0%)	22 (34.0%)	0.02	0.852
Education ≥ high school, *n* (%)	37 (58.0%)	38 (60.0%)	0.04	0.858
Body mass index, kg/m^2^	26.10 ± 3.20	26.30 ± 3.30	0.06	0.728
Current smoker, *n* (%)	25 (39.0%)	23 (36.0%)	0.06	0.715
Hypertension, *n* (%)	40 (63.0%)	38 (60.0%)	0.06	0.717
Diabetes, *n* (%)	17 (27.0%)	19 (29.0%)	0.04	0.694
NYHA class III–IV, *n* (%)	22 (34.0%)	20 (32.0%)	0.04	0.707
No. of stents, median (IQR)	1 (0,2)	1 (0,2)		0.99
Length of hospital stay, days	5.40 ± 1.70	5.60 ± 1.60	0.12	0.494

Values are mean ± SD unless otherwise indicated.SMD (standardized mean difference): for continuous variables, calculated using the pooled SD; for binary variables, calculated from group proportions. As a rule of thumb, |SMD| ≈ 0.10/0.20/0.50 can be interpreted as small/medium/large imbalance. *p*-values: Welch's *t*-test for continuous variables (except stents); *χ*^2^ test for categorical variables; Mann–Whitney *U* test for number of stents (SMD not computed for this row). All tests are two-sided. SMDs are reported regardless of statistical significance to aid assessment of baseline balance. SD, standard deviation; SMD, standardized mean difference; IQR, interquartile range; LVEF, left ventricular ejection fraction; SBP, systolic blood pressure; DBP, diastolic blood pressure; NYHA, New York Heart Association; HPLP-II, health-promoting lifestyle profile II; CADE-QSV, coronary artery disease education questionnaire—short version; SAQ, Seattle angina questionnaire.

**Table 3:** Comparison of CADE-QSV scores at different time points between the two groups (score, $\bar{x}\pm s$).

Outcome	Integrated *n* = 64	Routine *n* = 64	Mean difference(integrated -routine)	95% CI of difference	Welch t	*P*	Hedges’ g	95%CI for g
Baseline	13.68 ± 2.58	13.66 ± 2.55	+0.02	−0.88 to 0.92	0.05 (125)	0.962	0.01	−0.34 to 0.35
1 month	16.38 ± 1.89	14.89 ± 2.33	+1.49	0.75 to 2.23	3.97 (121)	<0.001	0.70	0.34 to 1.05
3 months	17.23 ± 1.77	15.88 ± 1.99	+1.35	0.69 to 2.01	4.05 (124)	<0.001	0.71	0.36 to 1.07
F	Fgroup = 55.764	Ftime = 101.231	Fgroup × time = 9.874					
*P*	<0.001	<0.001	<0.001					

Values are mean ± SD unless stated. Primary analysis used linear mixed-effects models (group, time, group × time) with Holm adjustment for multiple comparisons; per-timepoint Welch *t*-tests are shown as sensitivity analyses. Mean difference is Integrated -Routine. Hedges’ g is reported with 95% CI. Two-sided *α* = 0.05.

### 3.3 Comparison of SAQ scores at different time points between the two groups of patients

**Figure 3:**
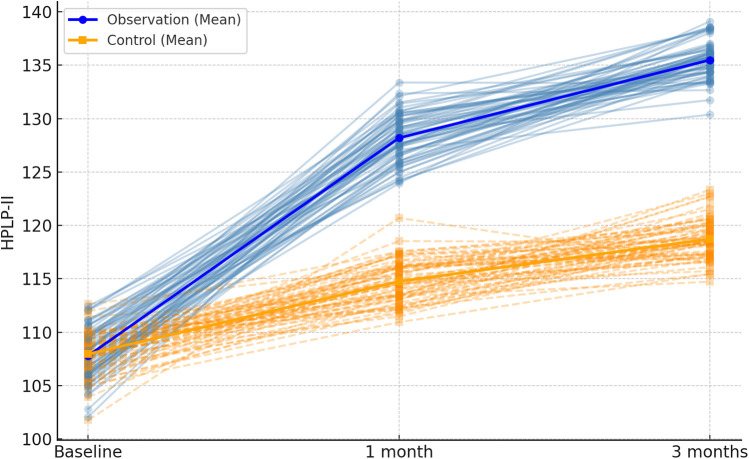
Spaghetti plot of SAQ total scores at each time point. The plot displays SAQ score trajectories for all patients. Blue lines = observation group; orange dashed lines = control group. Bold lines show average group scores over time.

Both the observation and control groups demonstrated an upward trend in SAQ scores after 1 and 3 months of intervention. Remarkably, the increase in SAQ scores within the observation group was significantly greater than that observed in the control group (*P* < 0.001), as outlined in Table 4; Figure 3.

**Table 4:** Comparison of SAQ scores at different time points between the two groups of patients (scores. $\bar{x}\pm s$).

Outcome	Integrated *n* = 64	Routine *n* = 64	Mean difference(integrated -routine)	95% CI of difference	Welch t	*P*	Hedges’ g	95% CI for g
Baseline	57.62 ± 8.33	57.89 ± 8.66	−0.27	−3.06 to 2.51	−0.19 (125.6)	0.853	−0.03	−0.38 to 0.31
1 month	70.23 ± 8.89	60.32 ± 5.74	+9.91	6.98 to 12.84	+6.30 (117.5)	<0.001	1.32	0.94 to 1.70
3 months	73.56 ± 9.11	63.45 ± 8.65	+10.11	6.99 to 13.24	+6.31 (124.0)	<0.001	1.13	0.76 to 1.50
F	Fgroup = 115.823	Ftime = 210.469	Fgroup × time = 14.376					
P	<0.001	<0.001	<0.001					

Values are mean ± SD unless stated. Primary analysis used linear mixed-effects models (group, time, group × time) with Holm adjustment for multiple comparisons; per-timepoint Welch *t*-tests are shown as sensitivity analyses. Mean difference is Integrated -Routine. Hedges’ g is reported with 95% CI. Two-sided *α* = 0.05.

### 3.4 Comparison of cardiac function indexes after PCI at different time points in the two groups

**Figure 4:**
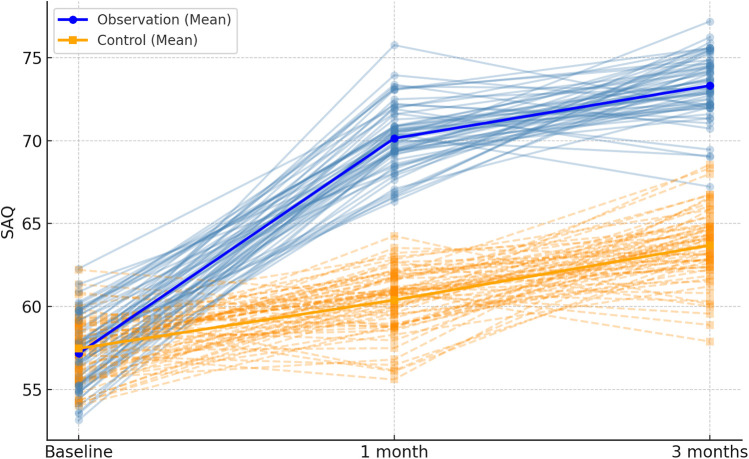
Spaghetti plot of left ventricular end-systolic diameter (LVESD) over time. Blue and orange lines represent individual changes in the observation and control groups, respectively. Group means are shown in bold.

**Figure 5:**
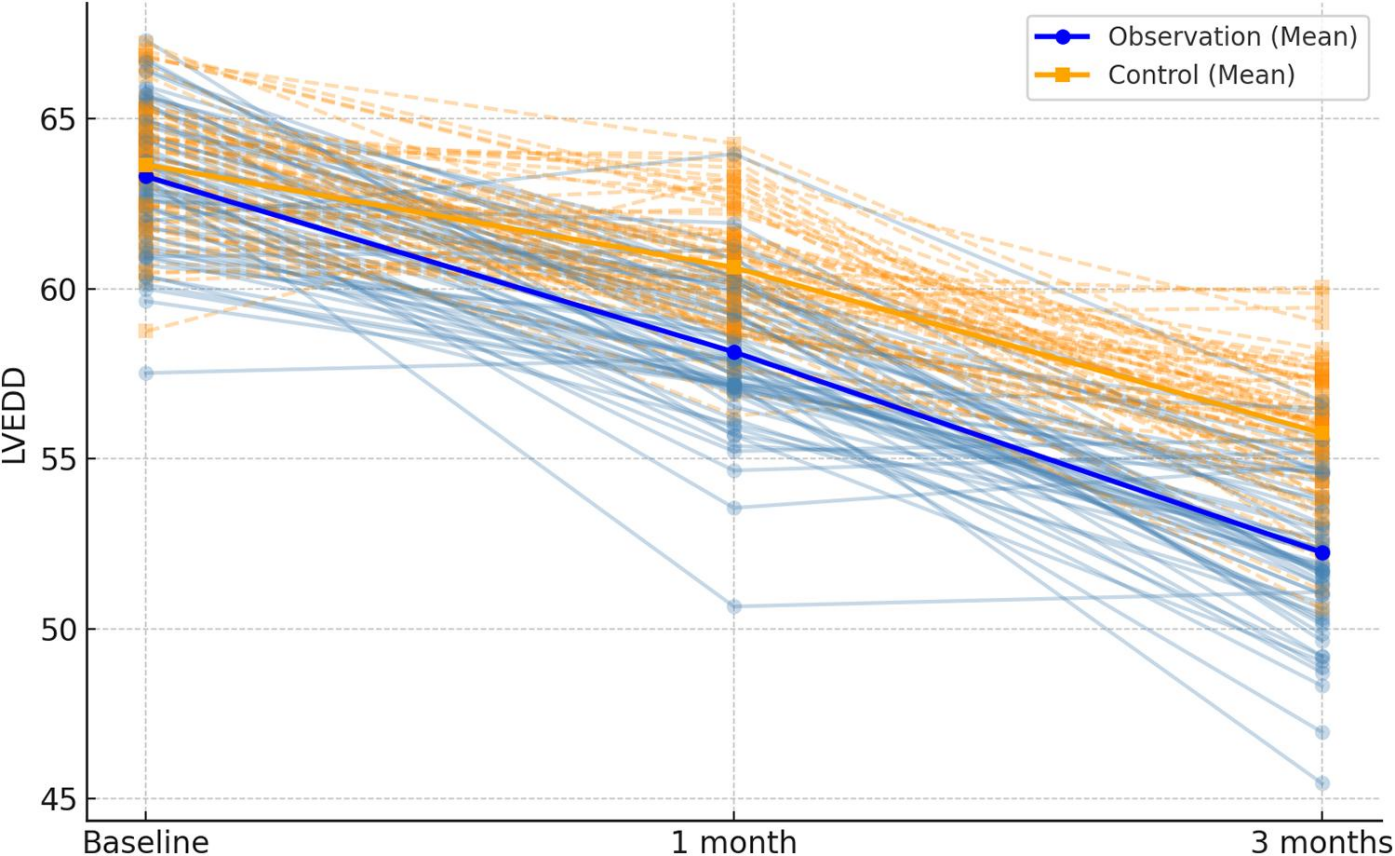
Spaghetti plot of left ventricular end-diastolic diameter (LVEDD) at three time points.trajectories reflect within-patient changes. Bold lines show group-level trends.

**Figure 6:**
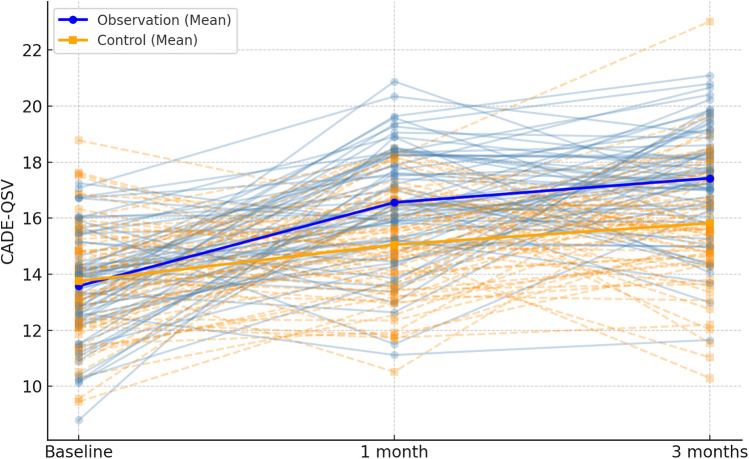
Spaghetti plot of left ventricular ejection fraction (LVEF) over time. The chart depicts improvements in cardiac function. Bold lines show average LVEF by group.

**Figure 7:**
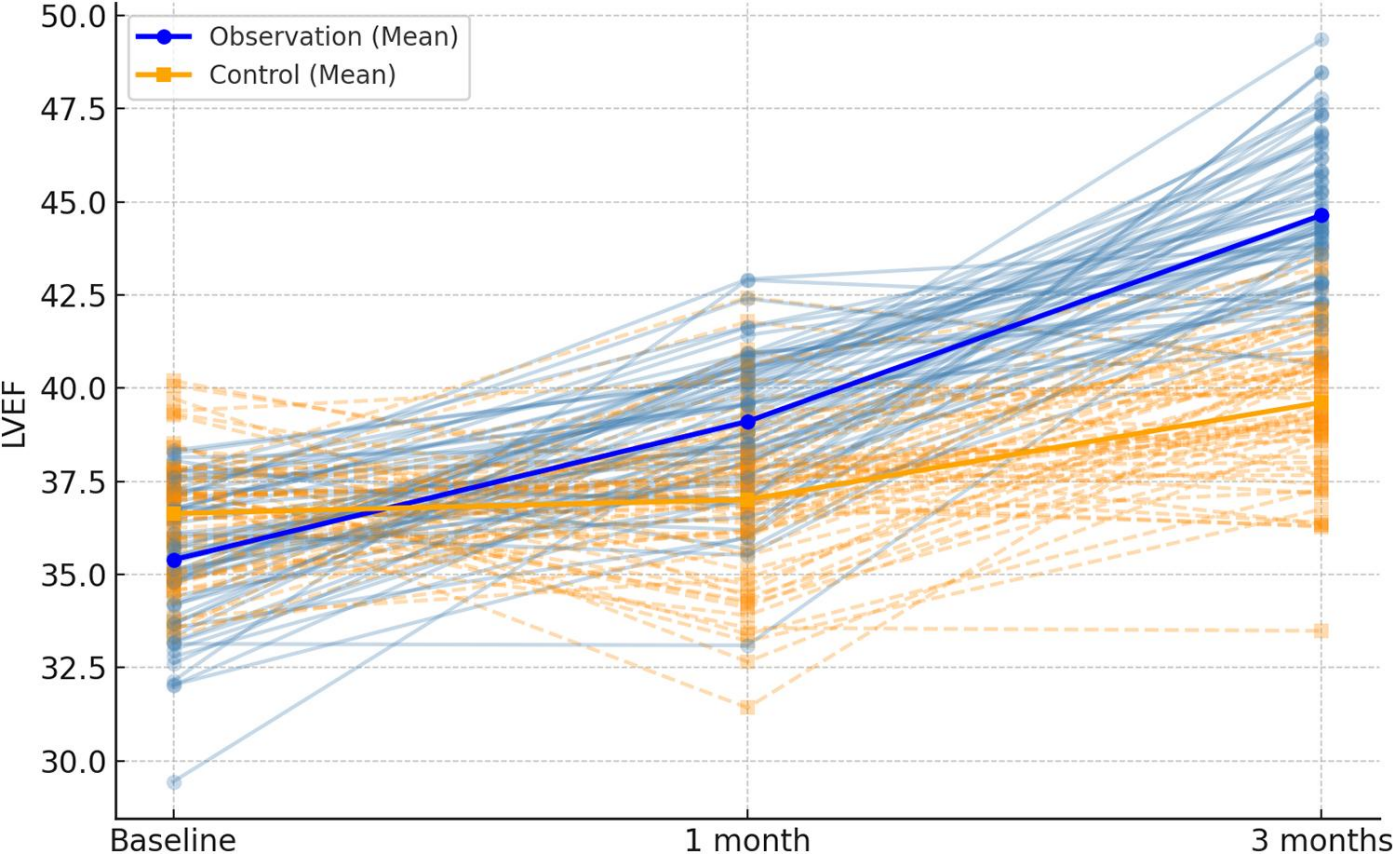
Spaghetti plot of left ventricular fractional shortening (LVFS) across time points. Lines represent individual patient changes. Group mean trajectories are highlighted.

Following 1 month and 3 months of intervention, both the observation and control groups exhibited a decreasing trend in left ventricular end-contraction internal diameter and left ventricular end-diastolic internal diameter. Notably, the decrease in these dimensions within the observation group was significantly more pronounced than that observed in the control group (*P* = 0.001, 0.005). Moreover, the left ventricular ejection fraction and left ventricular short-axis shortening rate in both groups displayed an increasing trend after 1 month and 3 months of intervention. Here again, the observation group demonstrated a more substantial increase compared to the control group (*P* < 0.001, = 0.001). These findings are summarized in Table 5; Figures 4–7.

**Table 5:** Comparison of cardiac function indexes after PCI at different time points in the two groups $\left(\bar{x}\pm s\right)$.

Outcome	LVESD (mm)		Mean difference(Integrated -Routine)	95% CI of difference	Welch t	*P*	Hedges’ g	95%CI for g
	Integrated *n* = 64	Routine *n* = 64						
Baseline	43.12 ± 6.22	43.99 ± 6.11	−0.87	−3.06 to 1.32	−0.80	0.426	−0.14	−0.49 to 0.21
1 month	38.36 ± 4.25	40.25 ± 5.59	−1.89	−3.59 to −0.19	−2.15	0.033	−0.38	−0.73 to 0.04
3 months	34.38 ± 6.77	38.56 ± 6.55	−4.18	−6.38 to −1.98	−3.55	0.001	−0.64	−1.00 to 0.29
F	Fgroup = 39.82	Ftime = 77.01	Fgroup × time = 8.42					
P	<0.001	<0.001	<0.001					
Baseline	63.38 ± 5.19	64.02 ± 5.23	−0.64	−2.52 to 1.24	−0.69	0.488	−0.12	−0.47 to 0.22
1 month	58.26 ± 4.23	60.52 ± 5.23	−2.26	−3.91 to −0.61	−2.69	0.008	−0.43	−0.78 to −0.08
3 months	52.63 ± 6.32	55.60 ± 6.32	−2.97	−4.88 to −1.06	−2.66	0.009	−0.50	−0.85 to −0.14
F	Fgroup = 35.97	Ftime = 88.54	Fgroup × time = 6.75					
P	<0.001	<0.001	<0.001					
Baseline	35.12 ± 4.13	36.22 ± 4.10	−1.10	−2.55 to 0.35	−1.51	0.133	−0.27	−0.62 to 0.08
1 month	39.23 ± 3.27	37.26 ± 3.38	+1.97	+0.79 to +3.15	+3.35	0.001	+0.59	0.24 to 0.94
3 months	44.26 ± 3.55	39.23 ± 3.52	+5.03	+3.72 to +6.34	+8.05	<0.001	+1.45	1.06 to 1.85
F	Fgroup = 91.53	Ftime = 127.36	Fgroup × time = 11.02					
P	<0.001	<0.001	<0.001					
Baseline	19.56 ± 2.33	19.08 ± 2.36	+0.48	−0.34 to +1.30	+1.16	0.249	+0.20	−0.15 to 0.55
1 month	21.36 ± 2.21	20.35 ± 2.12	+1.01	+0.26 to +1.76	+2.64	0.009	+0.46	0.11 to 0.81
3 months	23.89 ± 2.41	22.23 ± 2.43	+1.66	+0.84 to +2.48	+3.88	<0.001	+0.71	0.36 to 1.06
F	Fgroup = 79.45	Ftime = 110.29	Fgroup × time = 9.86					
P	<0.001	<0.001	<0.001					

Values are mean ± SD unless stated. Primary analysis used linear mixed-effects models (group, time, group × time) with Holm adjustment for multiple comparisons; per-timepoint Welch *t*-tests are shown as sensitivity analyses. Mean difference is Integrated -Routine. Hedges’ g is reported with 95% CI. Two-sided *α* = 0.05.

### 3.5 Comparison of changes in physiological indexes before and after intervention in the two groups

After 3 months, within-group improvements were significant in the observation group for diastolic blood pressure (*P* < 0.001), systolic blood pressure (*P* < 0.001), triglycerides (*P* < 0.001), and LDL (*P* < 0.001), whereas the change in BMI was not statistically significant (*P* = 0.057). In the control group, diastolic blood pressure (*P* = 0.0005), systolic blood pressure (*P* < 0.001), BMI (*P* = 0.017), triglycerides (*P* < 0.001), and LDL (*P* < 0.001) all showed significant pre-to-post differences. See Table 6.

**Table 6:** Comparison of changes in physiological indexes before and after intervention in the two groups $\left(\bar{x}\pm s\right)$.

Norm	Groups	Pre	3 months	*Δ* (3m−Pre)	Paired t (df = 63)	*P*
DBP ($\bar{x}\pm s$, mmHg)	Integrated *n* = 64	81.23 ± 12.23	75.23 ± 9.95	−6.00	−3.67	0.0005
	Routine *n* = 64	82.73 ± 12.29	78.16 ± 8.79	−4.57	−3.09	0.0030
SBP($\bar{x}\pm s$, mmHg)	Integrated *n* = 64	140.20 ± 12.39	132.68 ± 15.03	−7.52	−3.22	0.0020
	Routine *n* = 64	139.26 ± 18.62	120.23 ± 15.21	−19.03	−1.96	0.0546
BMI ($\bar{x}\pm s$, kg/m²)	Integrated *n* = 64	23.56 ± 3.77	23.36 ± 3.35	−0.20	−3.22	0.0020
	Routine *n* = 64	23.79 ± 3.56	23.36 ± 2.28	−0.43	−0.87	0.3882
TG ($\bar{x}\pm s$, mmol/L)	Integrated *n* = 64	4.29 ± 1.11	3.45 ± 0.79	−0.84	−7.49	<0.0001
	Routine *n* = 64	4.19 ± 1.05	3.31 ± 0.82	−0.88	−8.17	<0.0001
LDL ($\bar{x}\pm s$, mmol/L)	Integrated *n* = 64	2.47 ± 0.32	1.91 ± 0.33	−0.56	−17.78	<0.0001
	Routine *n* = 64	2.48 ± 0.38	1.89 ± 0.32	−0.59	−17.06	<0.0001

Values are mean ± SD. Within-group changes were tested using paired *t*-tests (df = 63). Two-sided *α* = 0.05.

### 3.6 Primary mixed-model results

**Figure 8:**
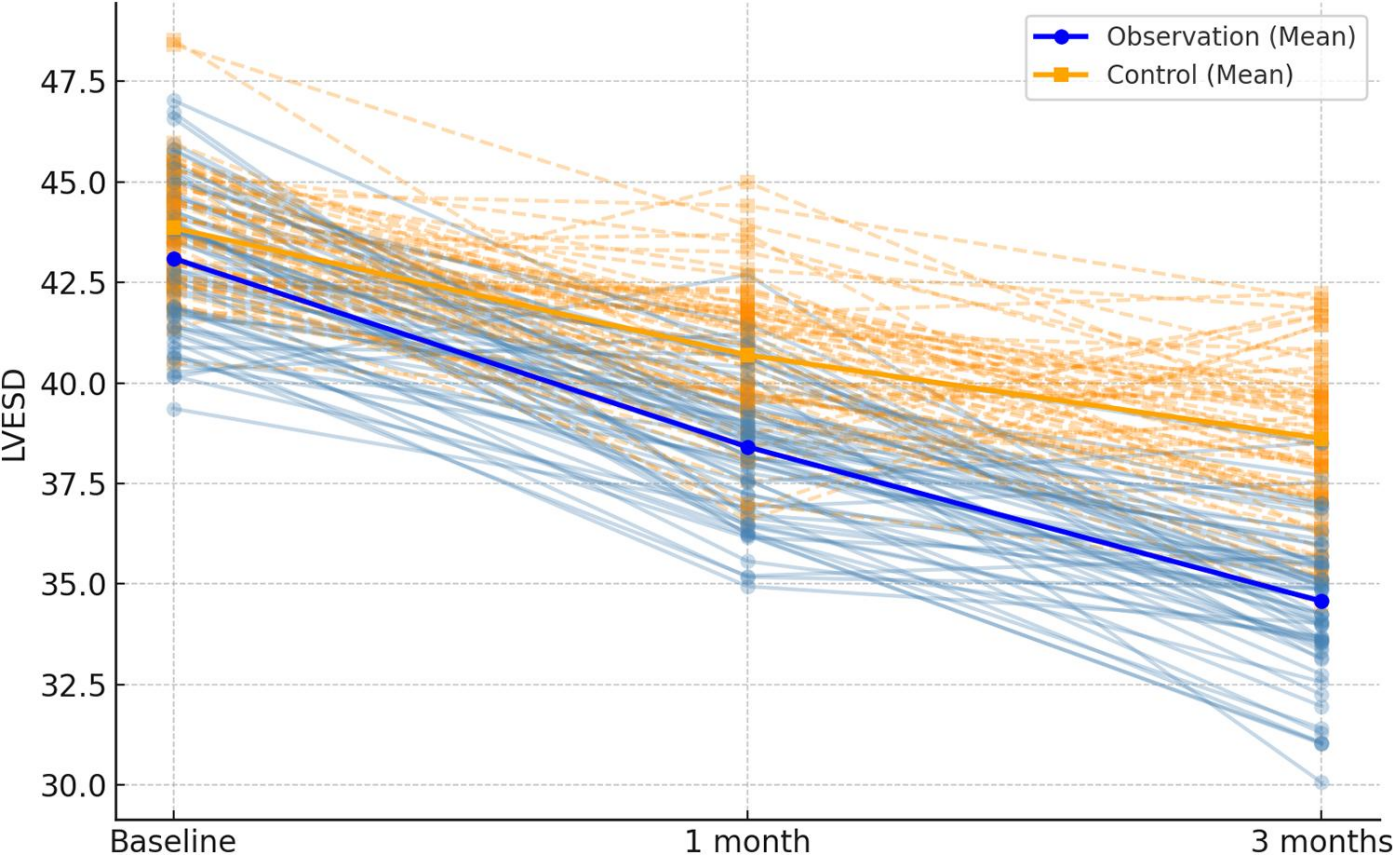
Combined spaghetti plot: trajectories of percent change from baseline (%) at baseline, 1 month, and 3 months (LVESD shown at baseline and 3 months only) for the integrated and routine groups. Eight lines share one axis: line style distinguishes groups (solid = Integrated; dashed = Routine), and markers distinguish outcomes (circle = SAQ-overall; square = HPLP-II; triangle = CADE-Q SV; diamond = LVESD). *Y*-axis: percent change from baseline; *X*-axis: time; *n* = 64 per group. Direction of benefit: higher is better for SAQ-overall, HPLP-II, and CADE-Q SV; lower is better for LVESD. SAQ, Seattle Angina Questionnaire; HPLP-II, Health-Promoting Lifestyle Profile II; CADE-Q SV, Coronary Artery Disease Education Questionnaire—Short Version; LVESD, left ventricular end-systolic diameter.

This table reports estimated marginal means (EMMs) at each time point, within-group change (*Δ* = 3-month−baseline), and the between-group difference-in-change (*ΔΔ* = *Δ*-observation−*Δ*_control) with 95% confidence intervals (CI) and Holm-adjusted *P* values (Holm-P). Each continuous outcome was modeled with an LMM including fixed effects for group (observation vs. control), time (baseline, 1-month, 3-month), and their interaction, and a subject-level random intercept; parameters were estimated by REML with Satterthwaite degrees of freedom. When baseline imbalance was present (SMD > 0.10 or *P* < 0.20), age, sex, LVEF, and NYHA class were prespecified covariates. Skewed biochemical variables (e.g., triglycerides) were log-transformed in modeling and, when appropriate, back-transformed for presentation. Multiplicity was controlled by Holm correction within prespecified families: (i) patient-reported outcomes (SAQ-overall, HPLP-II, CADE-Q SV); (ii) echocardiography (e.g., LVEF, LVFS, LVESD); (iii) physiological/biochemical markers (SBP, DBP, BMI, TG, LDL). The primary endpoint (3-month *ΔΔ* in SAQ-overall) is a single hypothesis and was not additionally adjusted; all tests are two-sided with *α* = 0.05. Directionality: *ΔΔ* is defined as Observation−Control; for outcomes where higher is better (SAQ-overall, HPLP-II, CADE-Q SV), *ΔΔ* > 0 favors the observation group, whereas for outcomes where lower is better (e.g., LVESD), *ΔΔ* < 0 favors the observation group. Sample size: *n* = 64 per group Table 7; Figure 8.

**Table 7:** Primary mixed-model results (linear mixed-effects model).

Family	Outcome	Integrated EMMs (Baseline → 3 m)	Routine EMMs (Baseline → 3 m)	*ΔΔ* (95% CI)	Holm-P
PROs	SAQ-overall	57.62 → 73.56 (*Δ* = +15.94)	57.89 → 63.45 (*Δ* = +5.56)	+10.38 (8.04∼12.72)	<0.001
PROs	HPLP-II	107.65 → 135.49 (*Δ* = +27.84)	108.23 → 119.23 (*Δ* = +11.00)	+16.84 (13.94 to 19.74)	<0.001
PROs	CADE-Q SV	13.68 → 17.23 (*Δ* = +3.55)	13.66 → 15.88 (*Δ* = +2.22)	+1.33 (0.69 to 1.97)	<0.001
Echo	LVESD (mm) ↓	43.12 → 34.38 (*Δ* = −8.74)	43.99 → 38.56 (*Δ* = −5.43)	−3.31 (−5.04 to −1.58)	<0.001
Physio	SBP (mmHg)	140.20 → 132.68 (*Δ* = −7.52)	139.26 → 120.23 (*Δ* = −19.03)	+11.51 (6.65 to 16.37)	<0.001
Physio	DBP (mmHg)	81.23 → 75.23 (*Δ* = −6.00)	82.73 → 78.16 (*Δ* = −4.57)	−1.43 (−4.91 to 2.05)	0.941
Physio	BMI (kg/m²)	23.56 → 23.36 (*Δ* = −0.20)	23.79 → 23.36 (*Δ* = −0.43)	+0.23 (−0.17 to 0.63)	0.902
Physio	Triglyceride (mmol/L)	4.29 → 3.45 (*Δ* = −0.84)	4.19 → 3.31 (*Δ* = −0.88)	+0.04 (−0.26 to 0.34)	0.989
Physio	LDL (mmol/L)	2.47 → 1.91 (*Δ* = −0.56)	2.48 → 1.89 (*Δ* = −0.59)	+0.03 (−0.06 to 0.12)	0.998

Values are estimated marginal means (EMMs) from linear mixed-effects models with fixed effects for Group, Time, and Group × Time and a subject-level random intercept. *Δ* denotes the model-based change from baseline to 3 months within each group; *ΔΔ* denotes the between-group difference-in-change (Integrated−Routine). 95% confidence intervals are based on Satterthwaite degrees of freedom. *P* values are Holm-adjusted within outcome families (patient-reported outcomes; echocardiography; physiological/biochemical). Transformations (e.g., log-TG) were applied as prespecified; when appropriate, estimates are back-transformed for presentation.

## 4 Discussion

This study provides empirical support for the effectiveness of an integrated nurse–physician management model in improving health behaviors, disease knowledge, cardiac function, and angina-related quality of life among post-PCI patients. In addition to statistical significance, the magnitude of improvements was clinically meaningful. At 3 months, SAQ-overall showed a between-group change of *ΔΔ* = +10.38 (95% CI: 8.04–12.72), and HPLP-II and CADE-QSV increased by *ΔΔ* = + 16.84 and *ΔΔ* = + 1.33, respectively—indicating substantial enhancements in lifestyle and disease understanding, which can support long-term recovery. The echocardiographic parameter LVESD changed by *ΔΔ* = −3.31, providing physiological confirmation of symptomatic improvements. These early benefits at 3 months highlight the practical utility of integrated care and underscore its potential for adoption in secondary prevention frameworks.

The study also enriches the empirical evidence on the role of integrated nurse–physician models in cardiovascular recovery. Recent nursing-focused studies have shown that nurse-led continuity of care and behavioral interventions significantly enhance post-PCI quality of life (14, 15). Moreover, structured WeChat-based interventions have been found to enhance medication adherence and disease knowledge in cardiac populations (16). Consistent with these findings, our results support the broader application of team-based, digitally augmented secondary-prevention strategies. Future research should extend follow-up to 6 or even 12 months to evaluate the durability of these benefits and incorporate cost-effectiveness analyses to inform policy implementation. cAlthough PCI can relieve vascular stenosis and restore blood flow, it does not eradicate the underlying atherosclerotic pathology. Studies report a 1-year recurrence rate of 20% to 60% post-PCI (17), making secondary prevention crucial. Our findings confirm that integrating nursing and medical specialties can more effectively foster health-promoting lifestyles in post-PCI patients. The traditional model of care is characterized by physician leadership with nurses in a supporting role, whereas integrated medical–nursing care is a multidisciplinary approach that leverages the strengths of different professionals and has demonstrated favorable effects in nursing education, clinical care, and research in recent years (18, 19). In this framework, our study shows that combining medical and nursing expertise helps foster the adoption of health-promoting lifestyles among patients with coronary artery disease after PCI. Behavioral improvements observed at 1 and 3 months corroborate this effect. The development of health behaviors entails awareness building, creating a supportive environment, motivating change, and sustaining action—processes that require joint effort by clinicians and patients over time. During the intervention, healthcare professionals jointly assessed individual needs, resources, and self-management capacity; activated patients’ engagement in behavior change; helped them abandon unhealthy habits while cultivating an enabling environment; provided health education to enhance disease knowledge and behavioral intent, thereby improving adherence; and used follow-up monitoring with timely feedback to facilitate the establishment and maintenance of healthy behaviors (20–23). Although integrated care programs have been disseminated across various disease populations, research specifically targeting lifestyle reconstruction in post-PCI patients remains limited. Our study demonstrated beneficial effects in this population. Future work should further capitalize on collaborative strengths and optimize intervention content and follow-up cadence to more comprehensively promote health behavior formation.In addition, we found that CADE-QSV scores increased in both groups at 1 and 3 months, with greater gains in the integrated-care group, suggesting that this model helps improve disease knowledge (the CADE-QSV instrument has sound reliability and validity). As the intervention progressed, the rate of knowledge gain slowed, indicating the need for longer follow-up to more fully delineate the trajectory of change. Meanwhile, SAQ scores improved at 1 and 3 months in both groups, with larger increases in the integrated-care group; echocardiographic parameters also shifted in favorable directions. Overall, these findings are consistent with prior evidence on improvements in quality of life and cardiac function (24, 25). Taken together, the results further support the integrated-care plus digital follow-up pathway as a clinically valuable strategy for post-PCI rehabilitation management. Though our findings are promising, several limitations must be acknowledged. The non-randomized, single-center observational design means residual confounding cannot be excluded. While adjustments were made for age, sex, LVEF, and NYHA class, time-varying covariates such as medication adherence, education level, or stent number were not modeled, which may bias results in either direction. Use of self-reported measures may introduce recall and social desirability biases. Although multiplicity adjustment was performed within outcome families, broader inferential control remains limited. Shared clinical pathways may pose contamination risks between groups, and generalizability is constrained by our specific tertiary hospital and cultural context in China. These issues underscore the need for prospective, multicenter studies with standardized adherence tracking and blinding strategies.

In summary, within 3 months after PCI, integrated nurse–physician management achieved greater improvements in patient-reported outcomes and cardiac function than routine care; in clinical practice, nursing strategies should be tailored to each patient's condition.

## Data Availability

The original contributions presented in the study are included in the article/Supplementary Material, further inquiries can be directed to the corresponding author.
